# Quality of life in patients with lower urinary tract symptoms associated with BPH: change over time in real-life practice according to treatment—the QUALIPROST study

**DOI:** 10.1007/s11255-015-1206-7

**Published:** 2016-01-25

**Authors:** Antonio Alcaraz, Joaquín Carballido-Rodríguez, Miguel Unda-Urzaiz, Rafael Medina-López, José L. Ruiz-Cerdá, Federico Rodríguez-Rubio, Darío García-Rojo, Francisco J. Brenes-Bermúdez, José M. Cózar-Olmo, Víctor Baena-González, José Manasanch

**Affiliations:** Urology Department, Hosp. Clínic Univ., IDIBAPS, Barcelona, Spain; Urology Department, Hosp. Univ. Puerta de Hierro, Majadahonda, Madrid Spain; Urology Department, Hosp. Univ. Basurto, Bilbao, Spain; Urology Department, Hosp. Univ. Virgen del Rocío, Sevilla, Spain; Urology Department, Hosp. Univ. La Fe, Valencia, Spain; Urology Department, Hosp. Univ. Puerto Real, Cádiz, Spain; Urology Department, Hosp. Univ. Parc Taulí Sabadell, Sabadell, Barcelona Spain; Llefià Primary Care Center, Badalona, Barcelona Spain; Urology Department, Complejo Hospitalario Universitario de Granada, Granada, Spain; Urology Department, Hosp. Univ. Carlos Haya, Málaga, Spain; Pierre Fabre Ibérica S.A., Ramon Trias Fargas, 7-11, 3º, 08005 Barcelona, Spain

**Keywords:** LUTS, BPH, Quality of life, Real-life practice, BII, IPSS

## Abstract

**Purpose:**

To evaluate change in quality of life (QoL) and symptoms in patients with lower urinary tract symptoms/benign prostatic hyperplasia (LUTS/BPH) in conditions of current clinical practice.

**Methods:**

Prospective, longitudinal, multicenter open-label study was carried out in urology outpatient clinics. Patients were ≥40 years of age with an International Prostate Symptom Score (IPSS) score ≥8. QoL and symptoms were measured at baseline and 6 months using the Benign Prostatic Hyperplasia Impact Index (BII) and the IPSS.

**Results:**

1713 patients were included for analysis. Mean (SD) IPSS and BII scores at baseline were 16.8 (5.4) and 6.8 (2.6), respectively. 8.9 % (*n* = 153) of study participants did not receive treatment (watchful waiting, WW), 70.3 % (*n* = 1204) were prescribed monotherapy (alpha-adrenergic blockers [AB]; phytotherapy [PT, of which 95.2 % was the hexanic extract of *Serenoa repens*, HESr]; or 5-alpha-reductase inhibitors [5ARI]), and 20.8 % (*n* = 356) received combined treatment (AB + 5ARI; AB + HESr; others). At 6 months, improvements in QoL were similar across the different medical treatment (MT) groups, both for monotherapy (AB: mean improvement [SD] of 2.4 points [2.4]; PT: 1.9 [2.4]; 5ARI: 2.5 [2.3]) and combined therapy (AB + 5ARI: 3.1 [2.9]; AB + PT: 3.1 [2.5]). There were no clinically significant differences between MT groups and all showed significant improvement over WW (*p* < 0.05). HESr showed similar efficacy to AB and 5ARI both as monotherapy and in combination with AB. Results on the IPSS were similar.

**Conclusions:**

Improvements in QoL and symptoms were equivalent across the medical treatments most widely used in real-life practice to manage patients with moderate or severe LUTS. HESr showed an equivalent efficacy to AB and 5ARI with fewer side effects.

## Introduction

Benign prostatic hyperplasia (BPH) is a common condition in older men that can often result in lower urinary tract symptoms (LUTS) [1]. LUTS associated with BPH (LUTS/BPH) can have a significant negative impact on patients’ quality of life (QoL) [2–4] as can certain treatments for the condition, some of which cause sexual dysfunction [5, 6].

Although the efficacy and safety of medical treatments such as alpha-adrenergic blockers, 5-alpha-reductase inhibitors, phytotherapy, combination therapy, antimuscarinic agents and phosphodiesterase type 5 inhibitors have been assessed in numerous clinical trials [7], fewer studies have evaluated those treatments in current clinical practice. Furthermore, observational studies to date have tended to focus on individual therapies, making it difficult to compare outcomes for different treatments under real-world conditions [8, 9]. There is therefore a need for large-scale studies which evaluate the range of treatments used to treat LUTS/BPH in daily practice and which allow results to be compared across treatments. Such studies are useful in that they provide complementary data to that obtained in controlled clinical trials, where patients, centers, and compliance may not be representative of broader clinical practice [10].

The Quality of Life in Benign Prostatic Hyperplasia (QUALIPROST) study was designed to assess change in the QoL of a large cohort of patients with moderate-to-severe LUTS/BPH managed using therapeutic approaches typically found in real-world clinical practice. Quality of life was assessed using the BPH Impact Index (BII), an international, validated questionnaire, and a further objective was to investigate how changes in symptoms correlated with changes in QoL.

## Subjects and Methods

### Patients and study design

This was a longitudinal, prospective, observational, multicenter study to evaluate change in QoL in patients with moderate-to-severe LUTS/BPH managed in a urological setting. The study was performed in centers throughout Spain from September 2009 to June 2011. Quality of life and BPH symptoms were measured at baseline and at a 6-month follow-up visit. Patients were included if they were ≥40 years of age with a diagnosis of LUTS/BPH and an IPSS score of ≥8. Patients were excluded if they had received drug treatment for BPH in the 6 months prior to inclusion or if they had received any drug treatment with a known effect on BPH symptoms (such as diuretics, antihistamines, or tricyclic antidepressants) for any length of time in the 4 weeks prior to inclusion. Patients were also excluded if they had other urinary disorders (prostatitis, urinary incontinence, urethral strictures, or prostate cancer) or if they had previously undergone surgery of the lower urinary tract.

### Study variables

The primary endpoint was change in QoL assessed using the validated Spanish version of the Benign Prostatic Hyperplasia Impact Index (BII), a self-administered questionnaire consisting of four questions measuring the impact of urinary symptoms on physical discomfort, worries about health, symptom bother, and interference with usual activities during the past month [11, 12]. Items are answered using a Likert scale, with four or five response options per item and scores range from 0 (best QoL) to 13 (worst QoL).

Symptoms of LUTS/BPH were evaluated using the validated Spanish version of the International Prostate Symptom Score (IPSS) [13]. Scores on this instrument range from 0 to 35 with a higher score indicating more severe symptoms and a change in IPSS score of ≥3.1 corresponding to a clinically meaningful change in patients’ global feeling of urination [14]. Both instruments were self-completed by patients at baseline and at the 6-month follow-up visit.

Sociodemographic data collected at baseline included age, weight, and height, the latter two being used to calculate the body mass index (BMI). Clinical data collected included date of initiation of urinary symptoms, year of LUTS/BPH diagnosis, and severity of BPH according to IPSS score (moderate = 8–19, severe = 20–35). We also collected data on diagnostic tests (digital rectal examination, prostate volume, Qmax, urine analysis, serum analysis, PSA), treatment received (yes/no, alpha-blockers, 5-alpha-reductase inhibitors, phytotherapy, other), and co-morbidities (high blood pressure, diabetes, dyslipidemia, or “other”), as well as treatment for co-morbidities. Side effects associated with treatment were recorded at the follow-up visit, and treatment compliance was assessed using the validated Spanish version of the Haynes–Sackett questionnaire [15]. This questionnaire consists of two parts. In the first part, the patient is asked whether he has difficulty taking his medication. In the second part, those who have answered yes to the first question are asked about the tablets they have taken in the previous month. Good adherence is considered to be achieved when the percentage of pills taken is between 80 and 110 % of the prescribed dose.

As this was a real-world study of patient management, investigators could prescribe any of the commercially available treatments according to their current practice. Based on treatment availability in Spain when the study was performed, several brands of alpha-blockers were prescribed. Tamsulosin was the most frequently prescribed alpha-blocker (principally Omnic^®^, Urolosin^®^ or generics, at a recommended dose [RD] of 0.4 mg daily), followed by finasteride (principally Proscar^®^ or generics at an RD of 5 mg/day). Only one brand of the following treatments was available and approved and covered by the NHS: dutasteride (Avidart^®^; RD: 0.5 mg/24 h), *P. africanum* (Tebetane compuesto^®^; RD: 60 mg/day), and hexanic extract of *Serenoa repens* (Permixon^®^; RD: 320 mg daily).

### Sample size

Sample size was calculated to detect a difference of 0.2 points in the BII overall score between baseline and follow-up with a statistical power of 80 % and a significance level of 0.05 using the Student *t* test for paired data. Assuming a loss to follow-up of 10 %, we calculated that a total sample size of 1638 patients would be required.

### Statistical analysis

Change over time within groups and differences in the size of change on the two primary outcome measures between groups receiving different medical treatments, or patients on watchful waiting, were assessed using parametric (Student *t* test) or nonparametric tests (Mann–Whitney) as appropriate. Analyses were carried out using per protocol (PP) and intent-to-treat (ITT) samples. All analyses were carried out for the overall study population and by subgroups categorized by medical treatment. Furthermore, a subset analysis was carried out in which patients were categorized by their baseline IPSS scores as moderate-low (8–13 points), moderate-high (14–19), and severe (≥20). As no differences in efficacy have been observed between the components of the therapeutic families of alpha-blockers and 5-alpha-reductase inhibitors, for the purposes of analysis in this study they were grouped together [16–18]. The correlation between change in symptoms as measured on the IPSS and change in QoL assessed using the BII overall score, was evaluated using Pearson’s correlation coefficient.

Comparisons of effectiveness between different medical treatments, and between medical treatment and watchful waiting, were carried out only after confirming that there were no statistically significant differences in baseline characteristics between the groups receiving different treatments. If this was not the case, results were offered in descriptive form. In all comparisons, results were considered statistically significant when *p* < 0.05. Statistical analyses were carried out using SAS 9.3 statistical software.

## Results

**Fig. 1:**
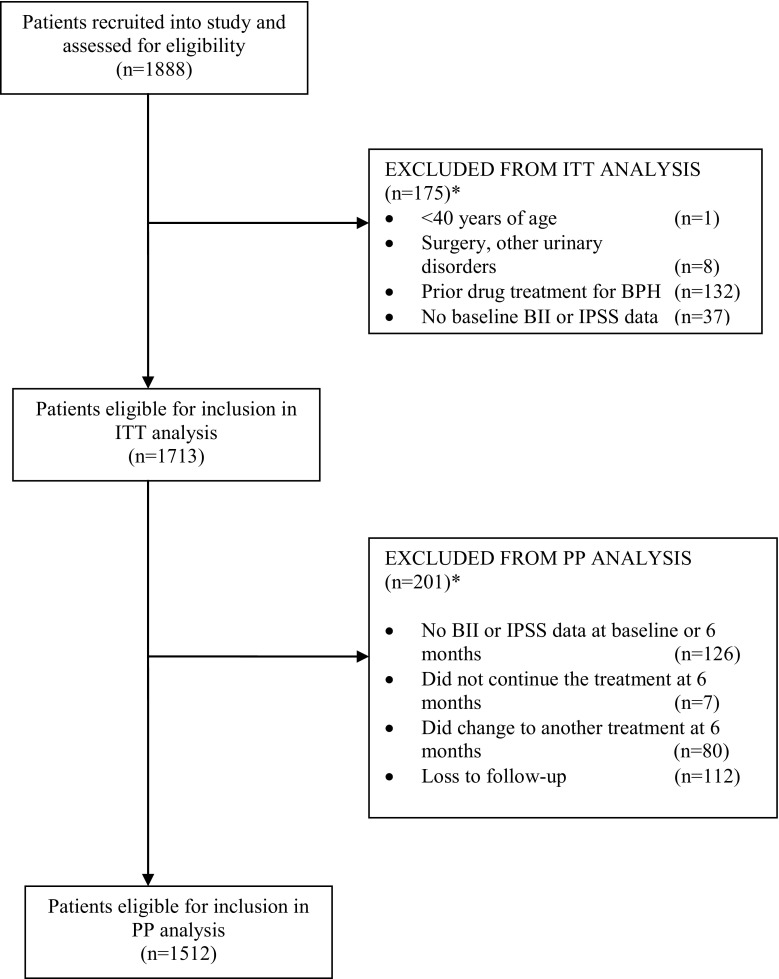
Flow diagram for patient inclusion in study. *Figures in individual rows may not sum to overall *n* as patients could have more than one exclusion criteria. *IPSS* International Prostate Symptom Score, *BII* Benign Prostatic Hyperplasia Impact Index, *ITT* Intention to treat, *PP* Per protocol

A total of 119 urologists participated in the study and a total of 1888 patients were recruited, of which 1713 were available for ITT analysis (Fig. 1). 6.5 % of patients were lost to follow-up. 11.1 % of patients included in the watchful waiting group switched to another treatment before study end. The proportion of patients switching treatments was very similar in the different pharmacological treatment groups (mean of 4.1 % across the groups).

Table 1 shows the study population’s sociodemographic and clinical characteristics according to treatment regimen. The mean (SD) time from diagnosis of BPH was 1.3 (2.8) years. In terms of treatment, 8.9 % of the patients were in WW, 70.3 % received monotherapy, and 20.8 % combined therapy. The number of patients initiating any type of treatment is shown in Table 1. Tamsulosin was the most frequently prescribed alpha-blocker (88.7 % of all alpha-blockers), dutasteride the most frequently prescribed 5ARI (53.2 % of all 5ARI), and hexanic extract of *S. repens* the most common phytotherapy (95.2 % of all phytotherapy).

**Table 1:** Patient characteristics at baseline; overall sample and by treatment regimen (ITT analysis)

Variable	Overall sample (*n* = 1713)	Watchful waiting (*n* = 153)	Monotherapy				Combination therapy			
			AB (*n* = 398)	5ARI (*n* = 94)	HESr (*n* = 678)	*P. africanum* (*n* = 34)	AB + 5ARI (*n* = 93)	AB + HESr (*n* = 219)	5ARI + HESr (*n* = 28)	Other (*n* = 16)
Age, mean (SD) years	64.0 (9.1)	63.1 (9.6)	64.7 (8.3)	69.3 (8.8)	61.7 (9.1)	63.7 (7.7)	69.4 (7.3)	64.6 (8.2)	71.0 (8.4)	67.5 (6.0)
BMI (kg/m^2^), mean (SD)	26.7 (2.9)	26.4 (2.4)	26.8 (2.8)	27.0 (2.6)	26.5 (2.9)	27.2 (2.7)	26.7 (2.8)	26.8 (3.0)	26.7 (2.3)	28.6 (3.1)
IPSS, mean (SD)	16.8 (5.4)	14.9 (5.2)	17.4 (4.8)	19.7 (5.6)	15.1 (4.9)	16.3 (5.6)	21.4 (5.8)	18.7 (5.4)	18.3 (5.9)	
BII, mean (SD)	6.8 (2.6)	5.9 (3.1)	7.3 (2.2)	7.8 (2.5)	6.0 (2.6)	6.3 (1.5)	8.3 (2.6)	7.9 (2.3)	7.2 (2.5)	
Qmax (mL/s), mean (SD)	12.9 (3.8)	13.5 (3.5)	11.7 (3.5)	12.6 (3.9)	14.0 (3.9)	12.5 (2.0)	10.8 (4.7)	12.8 (3.3)	11.1 (2.5)	13.7 (3.4)
Prostate volume (cm^3^), mean (SD)	51.1 (20.1)	46.7 (17.5)	52.2 (19.8)	68.9 (21.8)	43.3 (15.9)	55.5 (12.5)	72.7 (22.6)	53.2 (17.2)	69.9 (21.5)	59.9 (21.9)
Total PSA (ng/mL), mean (SD)	2.4 (1.3)	2.1 (1.2)	2.5 (1.3)	3.1 (1.5)	2.2 (1.3)	2.2 (1.0)	3.2 (1.3)	2.4 (1.4)	3.3 (1.5)	2.4 (1.1)

*AB* α- blockers, *5ARI* 5α-reductase inhibitors, *P. africanum* *Pygeum africanum, HESr* hexanic extract of *Serenoa repens,* *BMI* body mass index, *IPSS* International Prostate Symptom Score, *BII* Benign Prostatic Hyperplasia Impact Index, *PSA* prostate-specific antigen, *ITT* Intention to treat

Patients receiving phytotherapy tended to be slightly younger than patients in the other treatment groups. In terms of clinical characteristics, patients on watchful waiting (WW) and those treated with phytotherapy tended to have slightly lower baseline prostate volume and IPSS scores, and higher Qmax.

**Fig. 2:**
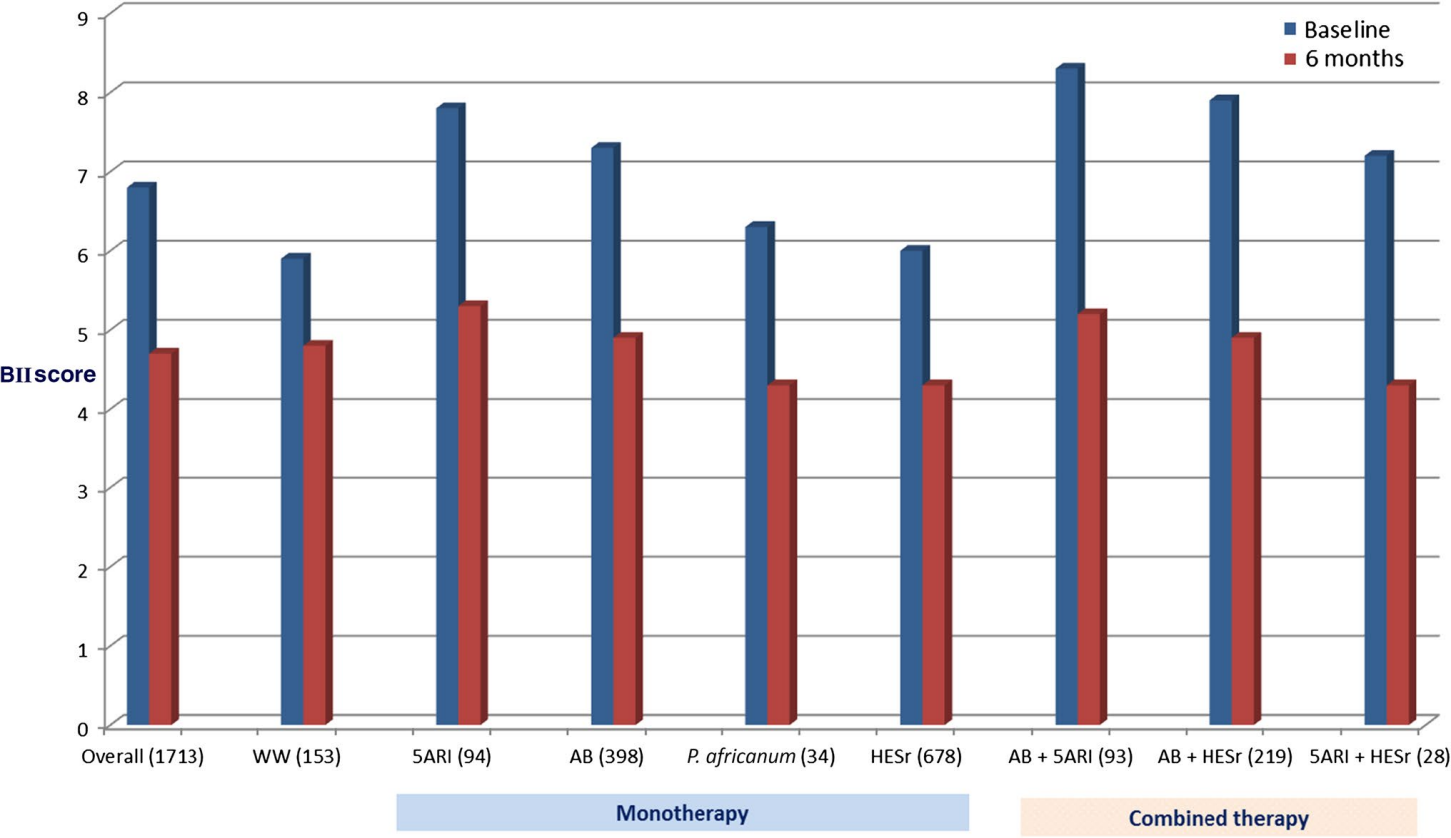
Baseline and end of study scores on BII, overall and by treatment group (*n* patients). *BII* Benign Prostatic Hyperplasia Impact Index. *WW* watchful waiting, *AB* α- blockers, *5ARI* 5α-reductase inhibitors, *P. africanum* *Pygeum africanum, HESr* hexanic extract of *Serenoa repens*

**Fig. 3:**
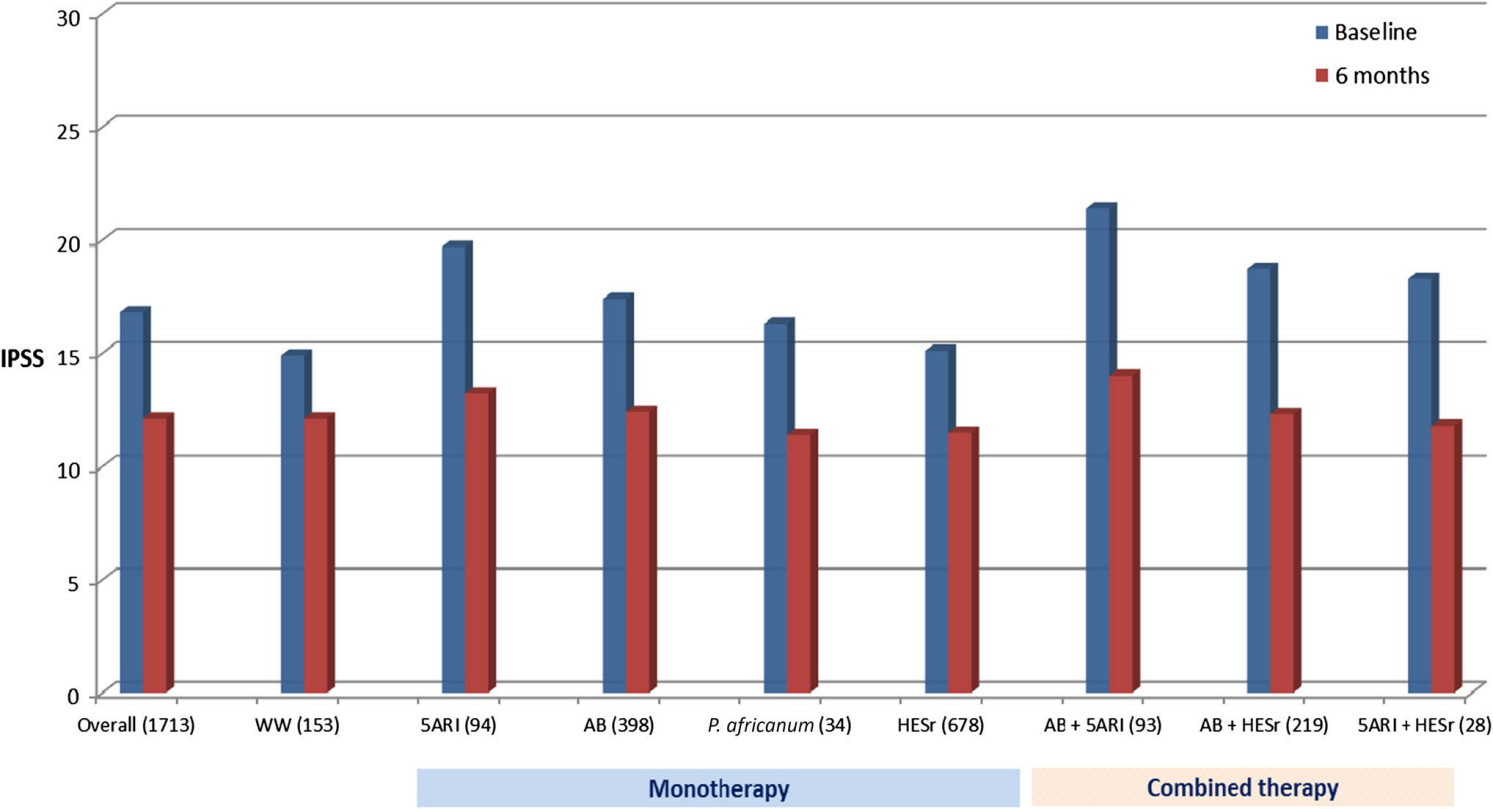
Baseline and end of study scores on IPSS, overall and by treatment group (*n* patients). *IPSS* International Prostate Symptom Score. *WW* watchful waiting, *AB* α-blockers; *5ARI* 5α-reductase inhibitors, *P. africanum* *Pygeum africanum*, *HESr* hexanic extract of *Serenoa repens*

Figures 2 and 3 show scores on the BII and IPSS, respectively, at baseline and at 6 months, overall and according to LUTS treatment. Patients receiving combination therapy had higher mean baseline BII and IPSS scores than those treated with monotherapy or WW. All medical treatment categories showed a relevant improvement in BII and IPSS scores after 6 months. The smallest improvement was observed in the WW group, with a mean (SD) change of 1.0 (2.2) and 2.5 (4.4) points on the BII and IPSS, respectively, compared to mean (SD) change scores of 2.3 (2.5) and 5.0 (4.9) for the same outcomes in treated patients.

When comparing monotherapy treatment overall with WW in patients with a baseline IPSS score between 14 and 19, a statistically significant mean (SD) improvement on IPSS score of 4.5 (3.7) was observed for monotherapy versus 3.0 (2.9) for WW (*p* < 0.006) and 2.2 (2.4) versus 1.1 (2.0) on the BII (*p* < 0.004). The largest changes in symptoms and QoL were observed in patients with more severe baseline symptoms (Tables 2, 3).

**Table 2:** Change scores on BII by treatment regimen according to baseline IPSS score

	Baseline IPSS (8–13)		Baseline IPSS (14–19)		Baseline IPSS (≥20)	
	*n*	Mean change	*n*	Mean change	*n*	Mean change
Watchful waiting, mean (SD)	64	0.3 (1.7)	46	1.1 (2.0)^1,2,3^		–
Monotherapy, mean (SD)						
AB	87	1.7 (1.9)	171	2.5 (2.4)^1,4^	118	2.8 (2.6)^7,8^
5ARI		–	34	2.1 (2.0)^2,4,5^	45	3.0 (2.5)^7,9^
HESr	269	1.2 (1.9)	256	2.0 (2.4)^3,5^	106	2.9 (3.1)^8,9^
Combination therapy, mean (SD)						
AB + 5ARI		–	25	2.3 (2.5)^6^	58	3.7 (3.0)^10^
AB + HESr	32	2.2 (2.5)	84	2.9 (2.4)^6^	86	3.7 (2.6)^10^

Groups with <25 patients were excluded from the analysis due to small sample size and a high degree of variability in results

Numbers in superscript indicate the results of statistical testing

If there is no superscript number, then it was not possible to test for significance due to differences in the mean IPSS baseline score

*IPSS* International Prostate Symptom Score, *BII* Benign Prostatic Hyperplasia Impact Index, *AB* α- blockers, *5ARI* 5α-reductase inhibitors, *HESr* hexanic extract of *Serenoa repens*

^1,2,3^Statistically significant difference observed at *p* < *0.05* between groups with the same superscript number

^4,5,6,7,8,9,10^No statistically significant differences observed between groups with the same superscript number

**Table 3:** Change scores on IPSS by treatment regimen according to baseline IPSS score

	Baseline IPSS (8–13)		Baseline IPSS (14–19)		Baseline IPSS (≥20)	
	*n*	Mean change	*n*	Mean change	*n*	Mean change
Watchful waiting, mean (SD)	64	0.5 (3.1)	46	3.0 (2.9)^1^		–
Monotherapy, mean (SD)						
AB	87	2.4 (2.6)	171	4.6 (3.5)^2,3^	118	7.6 (4.7)^5,6^
5ARI		–	34	5.2 (4.4)^3^	45	9.0 (6.0)^5,7^
HESr	269	1.7 (3.3)	256	4.2 (3.7)^1,2^	106	7.9 (5.3)^6,7^
Combination therapy, mean (SD)						
AB + 5ARI		–	25	4.4 (4.6)^4^	58	9.8 (6.5)
AB + HESr	32	2.8 (2.8)	84	5.0 (3.4)^4^	86	9.5 (5.1)

Groups with <25 patients were excluded from the analysis due to small sample size and a high degree of variability in results

Numbers in superscript indicate the results of statistical testing

If there is no superscript number, then it was not possible to test for significance due to differences in the mean IPSS baseline score

*IPSS* International Prostate Symptom Score, *BII* Benign Prostatic Hyperplasia Impact Index, *AB* α- blockers, *5ARI* 5α-reductase inhibitors, *HESr* hexanic extract of *Serenoa repens*

^1^Statistically significant difference observed at *p* < 0.05 between groups with the same superscript number

^2,3,4,5,6,7,8,9,10^No statistically significant differences observed between groups with the same superscript number

When changes in BII and IPSS scores were compared across groups receiving different monotherapies, taking into account initial symptom severity, no statistically significant differences were observed. In patients with moderate-high (14–19) or severe (≥20) baseline IPSS, all medical treatment modalities achieved a reduction in symptom severity of at least 4 points on the IPSS, which would represent a clinically significant improvement.

In patients with a baseline IPSS of 14–19, there were no statistically significant differences in the magnitude of improvement in QoL between patients treated with AB, 5ARI, or HESr as monotherapy when results were compared in two by two analyses. Likewise, in the same subgroup of patients, there were no statistically significance differences between AB and HESr or between AB and 5ARI in the magnitude of improvement on IPSS. Improvements in QoL in all three medical treatment groups (AB, 5ARI, HESr) were larger than those observed in the WW group, and all differences were significant (*p* < 0.05). Improvements in QoL and symptoms were similar in patients treated with AB + 5ARI or AB + HESr when baseline IPSS was taken into account, and no statistically significant differences were observed between these two groups.

In patients with more severe baseline symptoms (IPSS ≥ 20), improvements in QoL and IPSS scores were also similar between those treated with AB, 5ARI, or HESr and no statistically significant differences were observed between the groups in two by two comparisons of the treatments as monotherapy. Similar results, i.e., no statistically significant differences in the magnitude of improvement in QoL, were observed in the comparisons between AB + 5ARI and AB + HESr.

Changes on the IPSS and BII were highly correlated, with a coefficient of *r* = 0.66 in the study population overall (*p* < 0.0001). When analyzed by baseline symptom severity, correlations ranged from *r* = 0.60 (*p* < 0.0001) in the group with moderate baseline symptoms to *r* = 0.70 (*p* < 0.0001) in the group with severe baseline symptoms. A similar correlation was found between BII and question 8 (QoL) of the IPSS, at *r* = 0.57 for the overall sample (*p* < 0.0001), and *r* = 0.53 (*p* < 0.0001), and *r* = 0.56 (*p* < 0.0001) for the moderate, and severe baseline symptoms groups, respectively.

Table 4 shows the incidence of adverse effects (AE) overall and for the 5 most frequent reported AE. In patients receiving monotherapy, AB had the highest incidence of AE (16.3 %) and HESr the lowest (0.8 %); among combination therapies, AB + 5ARI had the highest rate of AE (30.5 %). Proportionally, the most frequent AE were erectile dysfunction and reduced libido. In terms of absolute numbers, retrograde ejaculation was the most common AE (31 patients in the AB group).

**Table 4:** Incidence of all-cause adverse effects after 6 months of follow-up

Treatment	*N*	Total AE, *n* (%)	Retrograde ejaculation	Reduced ejaculate volume	Erectile dysfunction	Reduced libido	Hypotension
Monotherapy, *n* (%)							
* AB*	424	69 (16.3)	31 (7.3)	19 (4.5)	3 (0.7)	4 (0.9)	10 (2.4)
* 5ARI*	106	15 (14.2)	0	2 (1.9)	10 (9.4)	9 (8.5)	0
HESr	733	6 (0.8)	0	0	0	0	0
* P. africanum*	34	1 (2.9)	0	0	0	0	0
Combination therapy, *n* (%)							
* AB* + *5ARI*	105	32 (30.5)	10 (9.5)	8 (7.6)	16 (15.2)	16 (15.2)	4 (3.8)
* AB* + HESr	234	33 (14.1)	12 (5.1)	12 (5.1)	7 (3.0)	2 (0.9)	6 (2.5)
* 5ARI* + HESr	29	5 (17.2)	0	1 (3.4)	3 (10.3)	3 (10.3)	0
* Other combinations*	20	3 (15)	0	1 (5.0)	3 (15)	1 (5.0)	0

*AE* adverse effects, *BP* blood pressure, *AB* α- blockers, *5ARI* 5α-reductase inhibitors, *P. africanum* *Pygeum africanum*, *HESr* hexanic extract of *Serenoa repens*

The PP analysis showed a similar pattern of results for all endpoints analyzed.

With respect to treatment compliance, approximately 90 % of patients in each medical treatment group (both monotherapy and combination treatment) reported that they had no difficulty taking the medication. Among those reporting some type of difficulty, 90 % of patients mentioned that they were taking >80 % of the prescribed treatment, except for 5ARI patients, who reported 80 %.

## Discussion

This study has evaluated changes in symptoms and QoL in a large cohort of patients with LUTS/BPH managed in conditions of real-life practice. We observed significant improvements in both symptoms and QoL in patients receiving any form of medical treatment. As could be expected, improvements were greater in patients with higher baseline IPSS scores and smaller in the WW group than in patients receiving any sort of medical treatment.

A relevant contribution of this study is that it assesses the effect on QoL of several medical treatments for LUTS/BPH used in real-life practice by means of an internationally recognized, validated questionnaire that is easy to use in regular clinical practice, whereas earlier studies tended to focus almost exclusively on symptoms [8, 18, 19] or on outcomes associated with a single drug [19, 20]. In the present study, treatment regimens were chosen by participating urologists based on their current practice, and the distribution of patients across different pharmacological options is in line with data published in a previous report [21].

All of the medical treatments studied were associated with improvements in both symptoms and QoL and the average improvement was similar to that observed in previous studies of different drug therapies using the BII and IPSS questionnaires [22–24]. Patients treated with hexanic extract of *S. repens* showed similar improvements in symptoms to those observed with AB or 5ARI monotherapy, thereby confirming the results of earlier randomized clinical trials [23–27]. It was not possible to compare IPSS outcomes between 5ARI and HESr in the group of patients with a moderate baseline IPSS (14–19) because of differences in baseline mean IPSS values.

Earlier publications have suggested that extracts of *S. repens* appear to be no more effective than placebo [28–30]; nevertheless, while not recommended by the AUA BPH Guideline, they are considered as a treatment option [31] and have well-established mechanisms of action [32–35]. It is important to note that general conclusions about *S. repens* can mask the fact that not all *S. repens* extracts have the same potency, and that the latter appears to be dependent on extraction procedure. Indeed, current European LUTS/BPH guidelines [36], while making no any recommendation on phytotherapy as a therapeutic group, do mention specific medications supported by clinical studies and a substantial weight of evidence regarding their efficacy. They also note that different brands of phytotherapy need to be assessed individually, as differences in their potency [36–39] mean that results from one brand cannot be extrapolated to another. In that sense, the results of clinical studies such as those cited above [28, 29] would only apply to the particular extract of *S. repens* used in those studies. The authors of the Cochrane Collaboration meta-analysis [30] came to a similar conclusion when they stated “we do not know if the present conclusions are generalizable to proprietary products of *S. repens* extracts, such as Permixon^®^ or Prostagutt^®^ forte.” A recent European Medicines Agency (EMA) report also concluded that only the hexanic extract of *S. repens* has sufficient evidence to support its use as a well-established medicinal product with recognized efficacy and acceptable safety [36].

As expected, adverse effects were lowest in patients treated with phytotherapy. On the other hand, almost 10 % of patients in the AB and 5ARI groups reported problems with sexual functioning, a proportion which is similar to previous reports [5, 40]. Specifically, treatment with alpha-blockers has been associated with a high incidence of ejaculatory disorder [41, 42], and a recent meta-analysis showed that ejaculatory dysfunction is significantly associated with the use of AB or 5ARI, with a threefold increase in risk for AB + 5ARI combination therapy compared with AB or 5ARI alone [43].

In the present 6-month study, combination therapy with AB + 5ARI and AB + HESr showed a similar level of improvement in BII score, though with a lower incidence of adverse effects for AB + HESr. The symptoms improvement measured by the IPSS questionnaire was also similar for these combinations, and not statistically significant differences were observed between the two therapeutic combinations in patients with a baseline IPSS score of 14–19. In patients with a baseline IPSS subgroup ≥20, though the improvement in symptoms was similar with both combinations, it was not possible to statistically compare outcome on the IPSS because of differences in mean baseline IPSS values. Previous studies have reported a tendency to use AB in combination with phytotherapy [44–46], but this is the first time to our knowledge that the effects of this combination on QoL and symptoms, and its tolerability, have been evaluated prospectively in real-life practice. Although improvement was equivalent between the two groups, the 6-month follow-up period did not allow us to draw any conclusions about disease progression. Nevertheless, progression appears to be slow and relatively limited in LUTS/BPH. Effectively, based on the MTOPS [47] and CombAT [48] trials, between 79 and 83 % of patients with moderate-severe LUTS/BPH would not be expected to show clinical progression after 4 years. In that case, intensive medical treatment may not offer more benefit than other treatments and could lead to more side effects [47, 48] causing a negative impact on QoL. It would be of interest to investigate more precise LUTS/BPH progression markers to more reliability identify patients who are likely to experience disease progression.

Finally, it is interesting to note that adherence in the present study was over 90 %, without major differences between the treatment groups in the present 6-months follow-up study. A recent retrospective study using data from an administrative prescription database [49] reported an adherence close to 65 % after 10 months in a broad population of patients receiving treatment for LUTS/BPH up to 8 years. However, the different methodologies used, different time periods, and the presence of other treatments in the analysis could explain the differences between the two studies.

The present study has some limitations. Data were obtained under conditions of real-life practice with no randomization or blinding; patients were therefore allocated to a specific management approach based on clinician judgment, which could lead to a selection bias. For example, patients treated with phytotherapy were younger and had less severe symptoms, which could explain some of the differences in outcomes because symptom relief and improvements in QoL are usually greater in patients with more severe symptoms [24]. This effect was minimized to some extent by grouping patients with similar baseline IPSS scores for analysis and confirming their comparability before analyzing the results. The relatively short follow-up period of six months could also be considered a limitation when studying a chronic disease. Nevertheless, it was not our intention to study disease progression and the study duration is in line with other recent studies, some of which used even shorter treatment periods [50, 51]. Finally, as this was an observational study in which we were interested in outcomes obtained under conditions of current clinical practice, there was no placebo arm. On the other hand, the inclusion of a watchful waiting group in this type of study can provide valuable information about the natural progression of the disease and what can be expected in patients who receive no treatment; in this case, the outcomes were notably better in all of the medical treatment groups than in the watchful waiting group.

Despite such limitations, real-world practice studies can contribute useful information on the outcomes associated with day-to-day patient management strategies and are a useful complement to clinical trials, the results of which do not always transfer to real-life practice [10]. In the present case, the large sample size also confers a high degree of precision and reliability on the results.

## Conclusions

Improvements in QoL and IPSS scores were equivalent across the medical treatments most widely used in real-life practice to manage patients with moderate or severe LUTS/BPH, and all medical treatments studied were associated with considerably larger improvements in QoL and symptoms than WW. Hexanic extract of *S. repens* showed equivalent efficacy to AB and 5ARI without the side effects on sexual function associated with those treatments, and its combination with AB appears to have a similar level of efficacy as the combination treatment with AB + 5ARI in the median term. The results of this study add to the evidence pool on current treatments for LUTS/BPH and should help to further inform decision-making regarding treatment. Such decision-making should also take into account the patient’s clinical condition and their risk–benefit preferences.

## Appendix

The following investigators participated in the QUALIPROST Study Group:

*A Coruña:* Dr. L. Busto Castañón; Dr. J.E. Duarte Novo; Dr. M. Montes Couceiro; Dr. A. Rodríguez Alonso; *Alicante:* Dr. J.A. Canovas Ivorra; Dr. C. López López; Dr. L. Prieto Chaparro; *Almería:* Dr. F. Gómez Berjón; Dr. J. Hortelano Parras; *Asturias:* Dr. A. M. Huescar; Dr. P.L. Muntañola Armora; *Badajoz:* Dr. E. Godoy Rubio; Dr. J. Mariño del Real; *Barcelona:* Dr. J. Armona Mani; Dr. F.J. Blasco Casares; Dr. S. Bucar Terrades; Dr. Ll. Cortadellas Angel; Dr. J. Fernández Zuazu; Dr. J.M. Malet Carreras; Dr. S. Mando Dakkar; Dr. J.M. Prats Puig; Dr. M. Puyol Pallás; Dr. J.A. Romero Martín; Dr. J. Sáenz de Cabezón Martí; Dr. J. Sánchez Macías; Dr. C. Vargas Blasco; Dr. J.M. Vayreda Martija; *Bilbao:* Dr. J.A. Gallego Sánchez; Dr. N. Prieto Ugidos; *Cádiz:* Dr. J.M. Arroyo Maestre; Dr. S. Garrido Insua; Dr. A. Gutiérrez de Pablo; Dr. M. Marmol Ruíz; Dr. F. Reyes Martínez; *Castellón:* Dr. J. Beltrán Persiva; Dr. L. Monzonis Rey; Dr. J. Palacios Castañeda; *Ciudad Real:* Dr. N. Jiménez López-Lucendo; Dr. D. Rodríguez Leal; *Córdoba:* Dr. J.C. Regueiro López; *Granada:* Dr. M. Cabezas Zamora; *Guipúzcoa:* Dr. G. Garmendia Olaizola; Dr. J.A. Rodríguez Andrés; *Jaén:* Dr. J. Jiménez Verdejo; Dr. E.J. Zarate Rodríguez; *León:* Dr. S.C. Gómez Cisneros; Dr. M. Lozano Rebollo; *Lleida:* Dr. J. Cortada Robert; Dr. L.M. Flavian Domenech; *Logroño:* Dr. A. Fernández Fernández; *Lugo:* Dr. F.J. Neira Pampin; *Madrid:* Dr. A. Abdallah Merhi; Dr. F. Arias Funes; Dr. I.T. Castillón Vela; Dr. J.M. Duarte Ojeda; Dr. M.J. García-Matres Cortés; Dr. J.F. Hermina Gutierrez; Dr. J.J. López-Tello García; Dr. C. Martín García; Dr. B. M.El-Awadeh; Dr. J.D. Rendón Sánchez; Dr. R. Rodríguez-Patron Rodríguez; Dr. J.C. Ruíz de la Roja; Dr. J. Vallejo Herrador; Dr. D. Vázquez Alba; *Málaga:* Dr. A. Bonilla Maldonado; Dr. J.M. Fernández Montero; Dr. A. Galacho Bech; Dr. C. Marchal Escalona; Dr. P. Rodero García; Dr. G. Sanz; Dr. J.D. Vázquez Cervilla; *Murcia:* Dr. G. Server Pastor; *Pontevedra:* Dr. D. Jamardo González; Dr. A. Selas Pérez; *Salamanca:* Dr. F. Díaz Alférez; Dr. M.F. Lorenzo Gómez; *Santander:* Dr. J.A. Portillo Martín; *Sevilla:* Dr. F.J. Giráldez Puig; Dr. M.A. Gutiérrez González; Dr. J.I. Huesa Martínez; Dr. Y. Ismail Tomaizeh; Dr. J. Leal Arenas; Dr. J. Martín Calero; Dr. J.L. Moyano Calvo; Dr. A. Ortiz Gámiz; Dr. J.M. Poyato Galán; Dr. J.A. Valero Puerta; Dr. E. Vilches Cocovi; *Tarragona:* Dr. P.L. Álvarez de la Red; Dr. J. Benagues Pamies; Dr. M. Prados Saavedra; *Tenerife:* Dr. T. Concepción Masip; Dr. C. Gómez de Segura Melcon; Dr. E. González de Chaves; Dr. J. Postius Robert; *Toledo:* Dr. F. Álvarez Fernández; Dr. A. Iglesias Justo; Dr. A. Melchor Galán; *Valencia:* Dr. J.E. Blasco Alfonso; Dr. M.A. Bonillo García; Dr. A. Collado Serra; Dr. J.A. García Cebrián; Dr. L.García Reboll; Dr. Y. Pallás Costa; Dr. M.J. Sánchez Sanchiz; Dr. J. Santamaría Meseguer; *Zaragoza:* Dr. A. García de Jalón Martínez; Dr. J.M. Gil Fabra; Dr. F. Monzón Alebesque; Dr. A. Ucar Terren.

## References

[1] Emberton M, Fitzpatrick JM, Garcia-Losa M, Qizilbash N, Djavan B (2008). Progression of benign prostatic hyperplasia: systematic review of the placebo arms of clinical trials. BJU Int.

[2] Welch G, Weinger K, Barry MJ (2002). Quality-of-life impact of lower urinary tract symptom severity: results from the Health Professionals Follow-up Study. Urology.

[3] Trueman P, Hood SC, Nayak US, Mrazek MF (1999). Prevalence of lower urinary tract symptoms and self-reported diagnosed ‘benign prostatic hyperplasia’, and their effect on quality of life in a community-based survey of men in the UK. BJU Int.

[4] Boyle P, Robertson C, Mazzetta C, Keech M, Hobbs R, Fourcade R (2003). The relationship between lower urinary tract symptoms and health status: the UREPIK study. BJU Int.

[5] Wu XJ, Zhi Y, Zheng J, He P, Zhou XZ, Li WB (2014). Dutasteride on benign prostatic hyperplasia: a meta-analysis on randomized clinical trials in 6460 patients. Urology.

[6] Rosen RC, Giuliano F, Carson CC (2005). Sexual dysfunction and lower urinary tract symptoms (LUTS) associated with benign prostatic hyperplasia (BPH). Eur Urol.

[7] Gravas S, Bach T, Bachmann A, Drake M, Gacci M, Gratzke C, et al (2015) Guidelines on the management of non-neurogenic male lower urinary tract symptoms (LUTS), incl. Benign Prostatic Obstruction (BPO) Uroweb. http://uroweb.org/wp-content/uploads/EAU-Guidelines-Non-Neurogenic-Male-LUTS-Guidelines-2015-v2.pdf. Accessed July 2015

[8] Batista JE, Palacio A, Torrubia R, Hernández C, Vicente J, Resel L (2002). Tamsulosin: effect on quality of life in 2740 patients with lower urinary tract symptoms managed in real-life practice in Spain. Arch Esp Urol.

[9] Vallancien G, Emberton M, Alcaraz A, Matzkin H, van Moorselaar RJ, Hartung R (2008). Alfuzosin 10 mg once daily for treating benign prostatic hyperplasia: a 3-year experience in real-life practice. BJU Int.

[10] Mishra V, Emberton M (2006). To what extent do real life practice studies differ from randomized controlled trials in lower urinary tract symptoms/benign prostatic hyperplasia?. Curr Opin Urol.

[11] Barry MJ, Fowler FJ, Oleary MP, Bruskewitz RC, Holtgrewe HL, Mebust WK (1995). Measuring disease-specific health status in men with benign prostatic hyperplasia. Measurement Committee of The American Urological Association. Med Care.

[12] Carballido Rodríguez J, Grunfeld Abellán A, Escudero Callen A, Bermejo GF, Regadera-Anechina L, Llach BX (2008). Validation of the Spanish version of the Benign Prostatic Hyperplasia Impact Index Questionnaire. “Validart Study”. Actas Urol Esp.

[13] Badía X, García-Losa M, Dal-Ré R, Carballido J, Serra M (1998). Validation of a harmonized Spanish version of the IPSS: evidence of equivalence with the original American scale. International Prostate Symptom Score. Urology.

[14] Barry MJ, Williford WO, Chang Y, Machi M, Jones KM, Walker-Corkery E (1995). Benign prostatic hyperplasia specific health status measures in clinical research: how much change in the American Urological Association symptom index and the benign prostatic hyperplasia impact index is perceptible to patients?. J Urol.

[15] Haynes RB, Sackett DL, Gibson ES, Taylor DW, Hackett BC, Roberts RS (1976). Improvement of medication compliance in uncontrolled hypertension. Lancet.

[16] Lukacs B, Cornu JN, Aout M, Tessier N, Hodée C, Haab F (2013). Management of lower urinary tract symptoms related to benign prostatic hyperplasia in real-life practice in France: a comprehensive population study. Eur Urol.

[17] Cindolo L, Pirozzi L, Fanizza C, Romero M, Sountoulides P, Roehrborn CG (2014). Actual medical management of lower urinary tract symptoms related to benign prostatic hyperplasia: temporal trends of prescription and hospitalization rates over 5 years in a large population of Italian men. Int Urol Nephrol.

[18] Hutchison A, Farmer R, Verhamme K, Berges R, Navarrete RV (2007). The efficacy of drugs for the treatment of LUTS/BPH, a study in 6 European countries. Eur Urol.

[19] Desgrandchamps F, Droupy S, Irani J, Saussine C, Comenducci A (2006). Effect of dutasteride on the symptoms of benign prostatic hyperplasia, and patient quality of life and discomfort, in clinical practice. BJU Int.

[20] Lukacs B, Grange JC, Comet D (2000). One-year follow-up of 2829 patients with moderate to severe lower urinary tract symptoms treated with alfuzosin in general practice according to IPSS and a health-related quality-of-life questionnaire. BPM Group in General Practice. Urology.

[21] Vallancien G, Pariente P (2001). Treatment of lower urinary tract symptoms suggestive of benign prostatic obstruction in real life practice in France. Prostate Cancer Prostatic Dis.

[22] Barkin J, Roehrborn CG, Siami P, Haillot O, Morrill B, Black L (2009). Effect of dutasteride, tamsulosin and the combination on patient-reported quality of life and treatment satisfaction in men with moderate-to-severe benign prostatic hyperplasia: 2-year data from the CombAT trial. BJU Int.

[23] Debruyne F, Koch G, Boyle P, Da Silva FC, Gillenwater JG, Hamdy FC (2002). Comparison of a phytotherapeutic agent (Permixon) with an alpha-blocker (Tamsulosin) in the treatment of benign prostatic hyperplasia: a 1-year randomized international study. Eur Urol.

[24] Debruyne F, Boyle P, Calais da Silva F, Gillenwater JG, Hamdy FC, Perrin P (2004). Evaluation of the clinical benefit of Permixon and tamsulosin in severe BPH patients—PERMAL study subset analysis. Eur Urol.

[25] Carraro JC, Raynaud JP, Koch G, Chisholm GD, Di Silverio F, Teillac P (1996). Comparison of phytotherapy (Permixon) with finasteride in the treatment of benign prostate hyperplasia: a randomized international study of 1,098 patients. Prostate.

[26] Al-Shukri SH, Deschaseaux P, Kuzmin IV, Amdiy RR (2000). Early urodynamic effects of the lipido-sterolic extract of *Serenoa repens* (Permixon(R)) in patients with lower urinary tract symptoms due to benign prostatic hyperplasia. Prostate Cancer Prostatic Dis.

[27] Boyle P, Robertson C, Lowe F, Roehrborn C (2004). Updated meta-analysis of clinical trials of *Serenoa repens* extract in the treatment of symptomatic benign prostatic hyperplasia. BJU Int.

[28] Bent S, Kane C, Shinohara K, Neuhaus J, Hudes ES, Goldberg H (2006). Saw palmetto for benign prostatic hyperplasia. NEJM.

[29] Barry MJ, Meleth S, Lee JY, Kreder KJ, Avins AL, Nickel JC (2011). Effect of increasing doses of Saw palmetto extract on lower urinary tract symptoms. JAMA.

[30] MacDonald R, Tacklind JW, Rutks I, Wilt TJ (2012). *Serenoa repens* monotherapy for benign prostatic hyperplasia (BPH): an updated Cochrane systematic review. BJU Int.

[31] AUA McVary KT, Roehrborn CG, Avins AL, Barry MJ, Bruskewitz RC, Donnell RF, Foster HE Jr, Gonzalez CM, Kaplan SA, Penson DF, Ulchaker JC, Wei JT. American Urological Association Guideline: Management of Benign Prostatic Hyperplasia (BPH) 2010, Reviewed and validity confirmed 2014. https://www.auanet.org/common/pdf/education/clinical-guidance/Benign-Prostatic-Hyperplasia.pdf. Accessed December 2015

[32] Bayne CW, Donnelly F, Ross M, Habib FK (1999). *Serenoa repens* (Permixon): a 5alpha-reductase types I and II inhibitor-new evidence in a coculture model of BPH. Prostate.

[33] Di Silverio F, Monti S, Sciarra A, Varasano PA, Martini C, Lanzara S (1998). Effects of long-term treatment with *Serenoa repens* (Permixon) on the concentrations and regional distribution of androgens and epidermal growth factor in benign prostatic hyperplasia. Prostate.

[34] Sirab N, Robert G, Fasolo V, Descazeaud A, Vacherot F, de la Taille A (2013). Lipidosterolic extract of *Serenoa repens* modulates the expression of inflammation related-genes in benign prostatic hyperplasia epithelial and stromal cells. Int J Mol Sci.

[35] Latil A, Libon C, Templier M, Junquero D, Lantoine-Adam F, Nguyen T (2012). Hexanic lipidosterolic extract of *Serenoa repens* inhibits the expression of two key inflammatory mediators, MCP-1/CCL2 and VCAM-1, in vitro. BJU Int.

[36] European Medicines Agency (2015) Committee on Herbal Medicinal Products (HMPC). Assessment report on *Serenoa repens* (W, Bartram) Small, fructus EMA/HMPC/137250/2013. Available at: http://www.ema.europa.eu/docs/en_GB/document_library/Herbal_-_HMPC_assessment_report/2014/12/WC500179593.pdf. Accessed June 2015

[37] Scaglione F, Lucini V, Pannacci M, Dugnani S, Leone C (2012). Comparison of the potency of 10 different brands of *Serenoa repens* extracts. Eur Rev Med Pharmacol Sci.

[38] Habib FK, Wyllie MG (2004). Not all brands are created equal: a comparison of selected components of different brands of *Serenoa repens* extract. Prostate Cancer Prostatic Dis.

[39] Scaglione F, Lucini V, Pannacci M, Caronno A, Leone C (2008). Comparison of the potency of different brands of *Serenoa repens* extract on 5alpha-reductase types I and II in prostatic co-cultured epithelial and fibroblast cells. Pharmacology.

[40] Giuliano F (2008). Medical treatments for benign prostatic hyperplasia and sexual function. BJU Int.

[41] Dutkiewics S (2001). Efficacy and tolerability of drugs for treatment of benign prostatic hyperplasia. Int Urol Nephrol.

[42] Novara G, Chapple CR, Montorsi F (2015). Individual patient data from registrational trials of silodosin in the treatment of non-neurogenic male lower urinary tract symptoms (LUTS) associated with benign prostatic hyperplasia (BPH): subgroup analyses of efficacy and safety data. BJU Int.

[43] Gacci M, Ficarra V, Sebastianelli A, Corona G, Serni S, Shariat SF (2014). Impact of medical treatments for male lower urinary tract symptoms due to benign prostatic hyperplasia on ejaculatory function: a systematic review and meta-analysis. J Sex Med.

[44] Fourcade RO, Lacoin F, Rouprêt M, Slama A, Le Fur C, Michel E (2012). Outcomes and general health-related quality of life among patients medically treated in general daily practice for lower urinary tract symptoms due to benign prostatic hyperplasia. World J Urol.

[45] Hizli F, Uygur MC (2007). A prospective study of the efficacy of *Serenoa repens*, tamsulosin, and *Serenoa repens* plus tamsulosin treatment for patients with benign prostate hyperplasia. Int Urol Nephrol.

[46] Ryu YW, Lim SW, Kim JH, Ahn SH, Choi JD (2015). Comparison of tamsulosin plus *Serenoa repens* with tamsulosin in the treatment of benign prostatic hyperplasia in Korean men: 1-year randomized open label study. Urol Int.

[47] McConnell JD, Roehrborn CG, Bautista OM, Andriole GL, Dixon CM, Kusek JW (2003). The long-term effect of doxazosin, finasteride, and combination therapy on the clinical progression of benign prostatic hyperplasia. N Engl J Med.

[48] Roehrborn CG, Siami P, Barkin J, Damião R, Major-Walker K, Nandy I (2010). The effects of combination therapy with dutasteride and tamsulosin on clinical outcomes in men with symptomatic benign prostatic hyperplasia: 4-year results from the CombAT study. Eur Urol.

[49] Cindolo L, Pirozzi L, Fanizza C, Romero M, Tubaro A, Autorino R, De Nunzio C, Schips L (2015). Drug adherence and clinical outcomes for patients under pharmacological therapy for lower urinary tract symptoms related to benign prostatic hyperplasia: population-based cohort study. Eur Urol.

[50] Kawabe K, Yoshida M, Homma Y, Silodosin Clinical Study Group (2006). Silodosin, a new alpha1A-adrenoceptor-selective antagonist for treating benign prostatic hyperplasia: results of a phase III randomized, placebo-controlled, double-blind study in Japanese men. BJU Int.

[51] Porst H, Kim ED, Casabé AR, Mirone V, Secrest RJ, Xu L (2011). Efficacy and safety of tadalafil once daily in the treatment of men with lower urinary tract symptoms suggestive of benign prostatic hyperplasia: results of an international randomized, double-blind, placebo-controlled trial. Eur Urol.

